# Risk score for predicting primary cesarean delivery in women with gestational diabetes mellitus

**DOI:** 10.1186/s12884-020-03306-y

**Published:** 2020-10-08

**Authors:** Chadakarn Phaloprakarn, Siriwan Tangjitgamol

**Affiliations:** Department of Obstetrics and Gynecology, Faculty of Medicine Vajira Hospital, Navamindradhiraj University, 681 Samsen Road, Dusit District, Bangkok, 10300 Thailand

**Keywords:** Gestational diabetes mellitus, Primary cesarean delivery, Risk score

## Abstract

**Background:**

Women with gestational diabetes mellitus (GDM) have a higher risk of cesarean delivery (CD) than glucose-tolerant women. The aim of this study was to develop and validate a risk score for predicting primary CD in women with GDM.

**Methods:**

A risk score for predicting primary CD was developed using significant clinical features of 385 women who had a diagnosis of GDM and delivered at our institution between January 2011 and December 2014. The score was then tested for validity in another cohort of 448 individuals with GDM who delivered between January 2015 and December 2018.

**Results:**

The risk score was developed using the features nulliparity, excess gestational weight gain, and insulin use. The scores that classified the pregnant women as low risk (0 points), intermediate risk (1–3 points), and high risk (≥ 4 points) were directly associated with the primary CD rates of the women in the development cohort: 14.7, 38.2 and 62.3%, respectively (*P* <  0.001). The model showed good calibration and acceptable discriminative power with a C statistic of 0.724 (95% confidence interval, 0.670–0.777). Similar results were observed in the validation cohort.

**Conclusion:**

A risk score using the features nulliparity, excess gestational weight gain, and insulin use can estimate the risk for primary CD in women with GDM.

## Background

The prevalence of gestational diabetes mellitus (GDM), one of the most common medical disorders of pregnancy, ranged from 1.8 to 25.1% in a previous report [1]. Its prevalence is increasing worldwide in parallel with the increased global prevalence of type 2 diabetes [2]. GDM poses multiple risks to pregnant women and their offspring, such as preeclampsia, macrosomia, and a consequent increase in obstetric interventions involving cesarean delivery (CD) [3, 4]. Several studies have reported that women with GDM have a higher risk of CD, specifically primary CD, than glucose-tolerant women [5–8]. One of the main reasons is to avoid complications associated with macrosomia, including shoulder dystocia and birth trauma. Although there are no current absolute indications for elective CD in women with GDM, standard practice guidelines recommend a scheduled CD when the estimated fetal weight (EFW) is 4500 g or more [9, 10].

Although a cesarean section is a common surgical procedure that can effectively prevent maternal and fetal mortality and morbidity when indicated, the complications of the procedure are well recognized, especially in an emergency setting, and include obstetric hemorrhage, postpartum infection, and the long-term effects of cesarean section scarring [11, 12]. Hence, the identification of women with GDM who are at risk of primary CD would be useful in clinical practice. For example, clinicians would be aware of the possibility of CD and arrange a better-prepared surgical team for women who require emergency CD to improve surgical outcomes.

Various characteristic features have been identified as risk factors for CD in women with GDM. These include advanced maternal age, nulliparity, obesity, gestational weight gain (GWG) above the Institute of Medicine (IOM) recommendations, and insulin use [6, 8, 13, 14]. In real clinical practice, an obstetrician may be reluctant to proceed with primary CD based on a patient’s individual risk. More solid evidence, obtained by combining these factors into a prediction model, would certainly be useful to support decision making. To date, there have been no studies that have focused on the development of a risk model to predict the likelihood of primary CD in women with GDM. The objective of this study was to develop and validate a risk score that can predict primary CD in women with GDM.

## Methods

### Development cohort

The risk score was developed in a development cohort comprising all pregnant women who attended our antenatal clinic, had a diagnosis of GDM, and delivered at our institution between January 2011 and December 2014. The exclusion criteria were women who had pregestational diabetes, multiple pregnancies, a history of previous uterine surgery (including previous CD and myomectomy), HIV infection, noncephalic presentation, or placental previa.

The sample size for the development of the risk scoring model was determined in accordance with the rule of thumb that a minimum of 10 outcome events are required per predicting factor [15]. We selected 3–5 common features of GDM individuals as predictive factors for primary CD. This would require a minimum of 50 women who had experienced the event to be recruited for the development of the prediction model.

Data from the development cohort were obtained from medical records. These included age, parity, prepregnancy body mass index, GWG, history of diabetes in any first-degree relatives, insulin use (type and dose), the presence or absence of primary CD, indications for primary CD, and neonatal birth weight. Prepregnancy body mass index was categorized into four groups based on the World Health Organization classification: underweight (< 18.5 kg/m^2^), normal (18.5–24.9 kg/m^2^), overweight (25.0–29.9 kg/m^2^) and obese (≥ 30 kg/m^2^) [16]. GWG was calculated as the difference in kilograms between the delivery weight and self-reported prepregnancy weight. We classified GWG as above or within/below the IOM recommendations. The recommended ranges of GWG are as follows: 12.5–18.0 kg for underweight women, 11.5–16.0 kg for women of normal weight, 7.0–11.5 kg for overweight women, and 5.0–9.0 kg for obese women [17].

According to our institutional guidelines for GDM screening, pregnant women with any risk factors underwent a glucose challenge test (GCT) at the first visit and were retested at 28–32 weeks of gestation if the first GCT value was normal. On the other hand, all other women without any risk were screened at 24–28 weeks of gestation. Any woman with an abnormal GCT was scheduled for a diagnostic 100-g oral glucose tolerance test. GDM was diagnosed according to the Carpenter and Coustan criteria [18]. Individuals with GDM were treated with diet control with or without insulin treatment based on their blood sugar levels and at the discretion of the endocrinologist. The indications for primary CD included labor dystocia, abnormal or indeterminate fetal heart rate (FHR) tracing, and elective CD due to an estimated large fetus, which was defined as an EFW above the 90th percentile for gestational age [19].

### Validation cohort

The risk score was tested in a validation cohort consisting of a different group of singleton pregnant women who attended our antenatal clinic and delivered at our institution between January 2015 and December 2018. The inclusion/exclusion criteria, data collection and risk scoring system for this cohort were the same as those used for the development cohort.

### Statistical analysis

Statistical analysis was performed with IBM SPSS Statistics for Windows, Version 22.0 (IBM Corporation, Armonk, NY, USA). Categorical variables are presented as numbers with percentages, and continuous variables are displayed as the medians with interquartile ranges. Categorical variables were compared by the chi-square test or Fisher’s exact test as appropriate. Mann-Whitney U test was used to compare continuous variables. Missing data were handled using multiple imputation. Binary variables were imputed by logistic regression (for two women in the development cohort with missing information about the history of diabetes in any first-degree relatives), whereas continuous variables were imputed by predictive means matching (for one woman in the development cohort and two women in the validation cohort with missing prepregnancy weights). All analyses were performed using imputed complete cases. A *P*-value < 0.05 was considered statistically significant.

The characteristic features of the development cohort that were associated with primary CD with a *P*-value < 0.20 in a univariate analysis were included in a multivariate logistic regression model. To develop a risk scoring system, we followed the methods previously described by Sullivan et al. [20]. In brief, the regression coefficient of each significant variable was divided by the lowest coefficient in the model before being rounded to the nearest integer to obtain the number of points. These score points were summed to derive a total risk score for each woman. The risks for primary CD were according to the total score into low risk, intermediate risk, and high risk.

The performance of the risk score was evaluated in terms of both discrimination and calibration. Discrimination (that is, the ability of the prediction model to differentiate between women who undergo primary CD and those who do not) was assessed using the C statistic or area under the receiver operating characteristic curve [21]. Calibration was evaluated by a calibration plot of observed probability versus predicted probability, which was grouped according to the quartile of predicted probability. The Hosmer-Lemeshow goodness-of-fit test was also used for calibration [22]. A value of *P >* 0.05 derived from this test was accepted as good calibration.

Ethical approval for the study was granted by the Vajira Institution Review Board (certificate of approval no.138/2561). This study is reported in line with the Transparent Reporting of a multivariable prediction model for Individual Prognosis Or Diagnosis (TRIPOD) statement [23].

## Results

### Development cohort

**Fig. 1:**
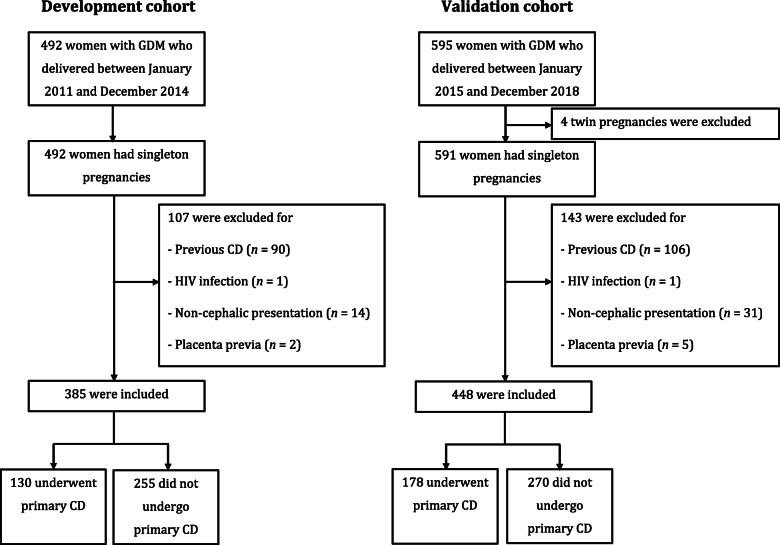
Enrollment of pregnant women. CD: cesarean delivery; GDM: gestational diabetes mellitus

From January 2011 to December 2014, 492 singleton pregnant women with GDM delivered at our institution. Of these, 107 women were excluded for certain reasons, as shown in Fig. 1. A total of 385 women were finally included in the development cohort.

The characteristic features of the women in the development cohort are presented in Table 1. The prevalence of primary CD in this cohort was 33.8%. The most common indication for primary CD was emergency CD due to labor dystocia (70.0%), followed by elective CD due to an estimated large fetus (23.8%) and emergency CD due to abnormal FHR tracing (6.2%).

**Table 1:** Characteristics of the women in the development and validation cohorts

Characteristic	Development cohort (*n* = 385)		*P*	Validation cohort (*n* = 448)		*P*
	Primary CD (Yes)	Primary CD (No)		Primary CD (Yes)	Primary CD (No)	
Number of women	130	255		178	270	
Age (years), median (IQR)	31.5 (27.0–35.0)	31.0 (27.0–36.0)	0.766	33.0 (28.0–36.0)	31.0 (27.0–35.0)	0.093
Age (years), *n* (%)			0.372			0.054
< 35	92 (70.8)	169 (66.3)		109 (61.2)	189 (70.0)	
≥ 35	38 (29.2)	86 (33.7)		69 (38.8)	81 (30.0)	
Parity, *n* (%)			< 0.001			< 0.001
Multiparity	45 (34.6)	180 (70.6)		57 (32.0)	179 (66.3)	
Nulliparity	85 (65.4)	75 (29.4)		121 (68.0)	91 (33.7)	
Prepregnancy BMI category (kg/m^2^), *n* (%)			0.301			0.478
Normal	74 (56.9)	150 (58.8)		83 (46.6)	139 (51.5)	
Underweight	7 (5.4)	26 (10.2)		18 (10.1)	31 (11.5)	
Overweight	33 (25.4)	51 (20.0)		51 (28.7)	60 (22.2)	
Obese	16 (12.3)	28 (11.0)		26 (14.6)	40 (14.8)	
Gestational weight gain, *n* (%)			0.003			< 0.001
Within/below IOM recommendations	78 (60.0)	190 (74.5)		105 (59.0)	214 (79.3)	
Above IOM recommendations	52 (40.0)	65 (25.5)		73 (41.0)	56 (20.7)	
History of diabetes in a first-degree relative, *n* (%)			0.271			0.992
No	90 (69.2)	190 (74.5)		126 (70.8)	191 (71.7)	
Yes	40 (30.8)	65 (25.5)		52 (29.2)	79 (29.3)	
Insulin use, *n* (%)			0.044			0.045
No	108 (83.1)	230 (90.2)		148 (83.1)	242 (89.6)	
Yes	22 (16.9)	25 (9.8)		30 (16.9)	28 (10.4)	
Type of insulin, *n* (%)						
Rapid-acting insulin	3 (2.3)	3 (1.2)	0.397	5 (2.8)	2 (0.7)	0.084
Short-acting insulin	8 (6.2)	7 (2.7)	0.102	6 (3.4)	3 (1.1)	0.095
Intermediate-acting insulin	8 (6.2)	2 (0.8)	0.002	6 (3.4)	10 (3.7)	0.853
Premixed insulin	11 (8.5)	19 (7.5)	0.726	17 (9.6)	20 (7.4)	0.420
Units of insulin used in pregnancy, median (IQR)	545 (256–1641)	456 (271–1622)	1.000	1029 (287–1747)	984 (387–2221)	0.785
Indication for primary CD, *n* (%)						
Elective CD due to an estimated large fetus	31 (23.8)	–	–	57 (32.0)	–	–
Emergency CD due to labor dystocia	91 (70.0)	–	–	112 (62.9)	–	–
Emergency CD due to abnormal FHR tracing	8 (6.2)	–	–	9 (5.1)	–	–
Neonatal birth weight (g), median (IQR)	3582 (3356–3882)	3108 (2845–3374)	< 0.001	3647 (3449–3880)	3062 (2821–3346)	< 0.001

*BMI* Body mass index, *CD* Cesarean delivery, *FHR* Fetal heart rate, *IOM* Institute of Medicine, *IQR* Interquartile range

Rates of nulliparity, GWG above the IOM recommendations, and insulin use were higher in the women who underwent primary CD than in the women who did not. These three features were identified as significant predictors of primary CD in both the univariate and multivariate analyses (Table 2). The regression coefficient values were 1.531 for nulliparity, 0.547 for GWG above the IOM recommendations, and 0.899 for insulin use. These coefficients were divided by 0.547 (the lowest value among the three) and then rounded to the nearest integer. The total score of each woman varied from 0 to 6 points (Table 2).

**Table 2:** Predictors of primary cesarean delivery determined from the data of the development cohort

Predictor	Crude OR (95% CI)	Adjusted OR^a^ (95% CI)	Coefficient	Points^b^
Parity				
Multiparity	1.00	1.00	Reference	0
Nulliparity	4.12 (2.27–7.47)	4.62 (2.91–7.36)	1.531	3
Gestational weight gain				
Within/below IOM recommendations	1.00	1.00	Reference	0
Above IOM recommendations	1.95 (1.23–3.06)	1.73 (1.06–2.81)	0.547	1
Insulin use				
No	1.00	1.00	Reference	0
Yes	1.87 (1.01–3.47)	2.46 (1.25–4.84)	0.899	2

*CI* Confidence interval, *OR* Odds ratio, *IOM* Institute of Medicine

^a^Adjusted for the other variables in the table. ^b^A point was allocated to each variable according to its coefficient value. Each coefficient was divided by 0.547 (the lowest coefficient among the significant predictors, corresponding to gestational weight gain above recommendations) and rounded to the nearest integer

**Fig. 2:**
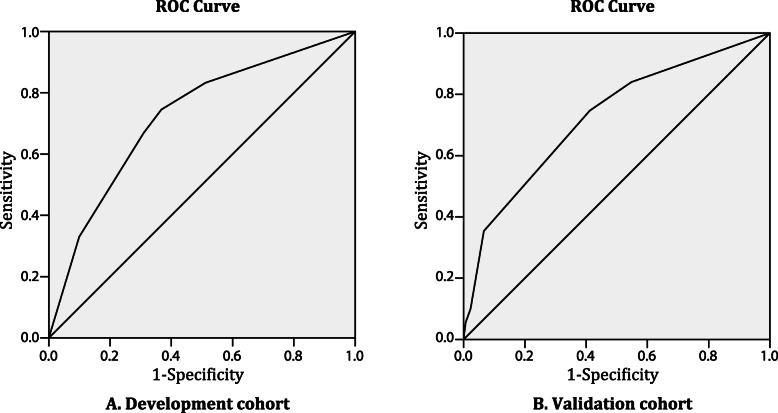
Receiver operating characteristic curves of the risk score for the prediction of primary cesarean delivery in the development (**a**) and validation (**b**) cohorts

**Fig. 3:**
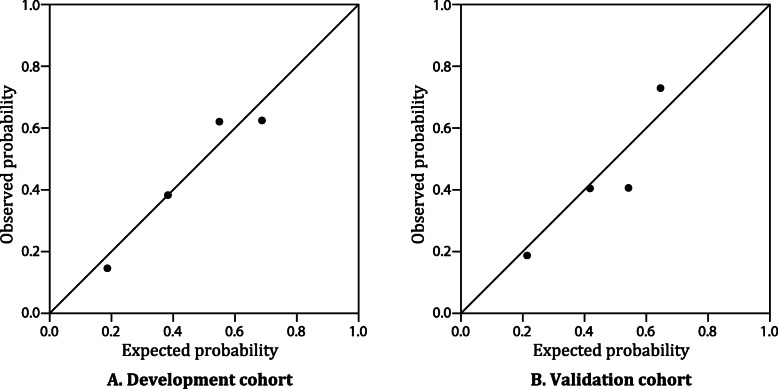
Calibration plot of the observed versus predicted probability of primary cesarean delivery for both the development (**a**) and validation (**b**) cohorts. The solid black line indicates the ideal calibration. The black dots indicate the observed frequencies by quartile of predicted probability

The receiver operating characteristic curve of the risk score yielded a C statistic of 0.724 (95% confidence interval, 0.670–0.777), indicating acceptable discriminatory power (Fig. 2). The calibration plot is presented in Fig. 3. The model displayed good calibration with no apparent under- or overprediction. The Hosmer-Lemeshow test demonstrated a good model fit, with a *P*-value of 0.929.

We classified the score points of the women in the development cohort into three categories to enhance the clinical utility of the risk model: low risk (0 points), intermediate risk (1–3 points), and high risk (≥ 4 points). This classification resulted in the assignment of 143 (37.2%) women to the low-risk category, 173 (44.9%) women to the intermediate-risk category, and 69 (17.9%) women to the high-risk category (Table 3). The rates of primary CD in the women were 14.7, 38.2, and 62.3%, respectively. The risk of primary CD was positively associated with the total score (*P* for trend < 0.001).

**Table 3:** Risk of primary cesarean delivery in the development and validation cohorts according to risk category

Risk category	Development cohort (*n* = 385)		Validation cohort (*n* = 448)	
	Total number of women	Number of women who underwent primary CD (%)	Total number of women	Number of women who underwent primary CD (%)
Low (0 points)	143	21/143 (14.7)	149	28/149 (18.8)
Intermediate (1–3 points)	173	66/173 (38.2)	218	87/218 (39.9)
High (≥ 4 points)	69	43/69 (62.3)	81	63/81 (77.8)

*CD* Cesarean delivery

### Validation cohort

From a database of 591 singleton pregnant women with GDM who delivered at our institution between January 2015 and December 2018, a total of 448 women who met the same inclusion/exclusion criteria as the development cohort were selected into the validation cohort (Fig. 1). Primary CD was observed in 39.7% of the women in this cohort. The significant factors associated with primary CD observed in the validation cohort were similar to those found in the development cohort (Table 1).

The C statistic of the risk score was 0.726 (95% confidence interval, 0.678–0.774) (Fig. 2). The calibration plot for the validation cohort showed reasonable calibration, as depicted in Fig. 3. The Hosmer-Lemeshow *P*-value was 0.250, indicating good calibration.

When the categorization of the score points was applied to the validation cohort, the results were similar to those for the development cohort: 149 (33.2%) women were assigned to the low-risk category, 218 (48.7%) women to the intermediate-risk category, and 81 (18.1%) women to the high-risk category (Table 3). There was a positive association of the primary CD risk with the risk category (*P* for trend < 0.001). The primary CD rates for the low-, intermediate-, and high-risk categories were 18.8, 39.9, and 77.8%, respectively (Table 3).

## Discussion

Primary CD, both elective and emergency CD, is commonly encountered in women with GDM. Current international practice guidelines recommend elective CD in women with GDM who have an EFW of 4500 g or more in order to reduce complications from difficult vaginal delivery [9, 10]. Aside from elective CD, emergency CD is also increased in GDM due to a higher chance of abnormal FHR patterns compared to glucose-tolerant women [24]. Our study focused on these two conditions of large fetuses and abnormal FHR tracings in an assessment of unfavorable impacts of GDM.

We developed and externally validated a simple prediction model for primary CD in two different groups of women with GDM (development and validation cohorts) who attended our antenatal clinic under similar conditions. The proposed risk score showed acceptable discriminatory power (C statistic of 0.724) and was clearly supported by external validation (C statistic of 0.726). The Hosmer-Lemeshow test indicated good calibration without a difference of predictive power between the development and validation cohorts (*P* > 0.05). To our knowledge, this is the first risk scoring system to be developed and validated for the identification of primary CD in individuals with GDM using only basic clinical data.

The three risk factors for primary CD in our risk scoring system, in descending order of importance, were nulliparity, insulin use, and GWG above the IOM recommendations. These three variables were also identified as factors that were significantly associated with primary CD in previous studies [8, 13, 14]. The adjusted odds ratio of 4.62 for nulliparity as a risk factor for primary CD that was demonstrated in our study was comparable to the finding of Gascho et al. [13], who also reported a 4.6-fold-greater odds of primary CD for nulliparous pregnant women with GDM. Likewise, the 1.46-fold risk of primary CD among the women who were treated with insulin (108/338 women or 46.8%) compared to the women without insulin treatment (22/47 women or 32.0%) observed in our study was similar to the relative risk of primary CD of 1.48 among insulin-treated pregnant women with GDM (24.7%) compared to those without insulin therapy (16.7%) found in the study of Ehrenberg et al. [8]. Regarding GWG, Cheng et al. [14] found that the risk of primary CD was increased by 1.52 fold among individuals with GDM whose weight gain exceeded the IOM guidelines compared to women with a GWG within the guidelines. This odds ratio was slightly lower than the odds ratio of 1.73 observed in the present study, which compared women with a GWG above the IOM recommendations with those whose GWG was within/below the recommendations.

Of the three identified risk factors for primary CD, GWG above the IOM recommendations appeared to be a modifiable risk factor. Preventive measures to reduce the likelihood of excessive GWG may lower the risk of primary CD. This goal could be achieved through a strict diet and a planned target weight gain throughout pregnancy [25–27].

Our risk score had a moderate degree of accuracy for classifying women with GDM into low, intermediate, and high risk categories for primary CD. In the development cohort, 43 out of 69 women who were classified as being at high risk for primary CD ended up having CD, giving a positive predictive value of 62.3% and a negative predictive value of 72.5%. In the validation cohort, the positive predictive value and negative predictive value of the risk score for primary CD among women who were classified into the high-risk category were 77.8 and 68.7%, respectively. Categorizing individuals with GDM into different risk categories would allow clinicians to thoroughly inform pregnant women about their estimated risk of primary CD. This could also alert attending staff for the proactive early detection of intrapartum conditions that lead to CD, including labor dystocia and abnormal FHR tracing, in women at high risk.

We were aware of a few limitations of the study. Being a retrospective study, some data that might contribute to the risk of primary CD were not available or incomplete, for example, glucose control status, the presence or absence of polyhydramnios, fetal abdominal circumference, and EFW. However, we believe that the small number of features (with satisfactory results) certainly makes the risk tool simpler for clinical application. Further, the rule of thumb for sample size calculation was unable to consider the magnitude of predictor effects and distribution of predictors. Hence, the clinicians who would apply this in clinical practice must be aware of this limitation. In addition, the points for each variable were converted from the original regression coefficients to integers. This conversion could lead to some loss in predictive accuracy. Another limitation was the similar characteristics of the development and validation cohorts (that is, pregnant women seeking medical care at the same hospital and country). Additional external validation using cohorts in different settings is required to confirm the performance and generalizability of the model. Last, although the risk score can help in predicting the risk of CD, it can also lead to maternal anxiety. Hence, clinicians should realize its advantages and disadvantages when applying the risk score in clinical practice.

Our study has several strengths. First, the sample size of the development cohort was adequate to build a reliable and robust model. Second, a multiple imputation technique was applied to handle missing data to maintain the statistical power and unbiased results. Third, the sample size of the validation cohort was also adequate (meeting the requirement of a minimum of 100 events and/or 100 nonevents) [28] to support its generalizability. The concordant results between the development and validation cohorts suggested the reliability of the model. Last, the risk score is a novel but simple approach that includes only basic characteristic features of pregnant women and uses a points system that can readily estimate the risk at a glance.

## Conclusion

We developed a new risk score using three characteristic features to predict primary CD in women with GDM. The score was well validated with high reliability. Risk stratification based on the score could help clinicians assess the chance of primary CD in each woman with certainty. The application of the model should guide attending staff in closely monitoring women at high risk for primary CD. Proactive early detection with the management of some modifiable risk factors, through, for example, strict dietary and weight control, may reduce the risk of primary CD in women with GDM and improve obstetric outcomes.

## Data Availability

The datasets used and analyzed in this study are available from the corresponding author on reasonable request.

## Abbreviations

CD: Cesarean delivery
EFW: Estimated fetal weight
FHR: Fetal heart rate
GCT: Glucose challenge test
GDM: Gestational diabetes mellitus
GWG: Gestational weight gain
HIV: Human immunodeficiency virus
IOM: Institute of Medicine
TRIPOD: Transparent Reporting of a multivariable prediction model for Individual Prognosis Or Diagnosis
